# Quantifying and visualizing site performance in clinical trials

**DOI:** 10.1016/j.conctc.2018.01.005

**Published:** 2018-01-31

**Authors:** Eric Yang, Christopher O'Donovan, JodiLyn Phillips, Leone Atkinson, Krishnendu Ghosh, Dimitris K. Agrafiotis

**Affiliations:** Covance Inc., 210 Carnegie Center, Princeton, NJ 08540, USA

**Keywords:** Data visualization, Site performance, Investigator performance, Clinical trial optimization, Alzheimer's disease

## Abstract

**Background:**

One of the keys to running a successful clinical trial is the selection of high quality clinical sites, i.e., sites that are able to enroll patients quickly, engage them on an ongoing basis to prevent drop-out, and execute the trial in strict accordance to the clinical protocol. Intuitively, the historical track record of a site is one of the strongest predictors of its future performance; however, issues such as data availability and wide differences in protocol complexity can complicate interpretation. Here, we demonstrate how operational data derived from central laboratory services can provide key insights into the performance of clinical sites and help guide operational planning and site selection for new clinical trials.

**Methods:**

Our methodology uses the metadata associated with laboratory kit shipments to clinical sites (such as trial and anonymized patient identifiers, investigator names and addresses, sample collection and shipment dates, etc.) to reconstruct the complete schedule of patient visits and derive insights about the operational performance of those sites, including screening, enrollment, and drop-out rates and other quality indicators. This information can be displayed in its raw form or normalized to enable direct comparison of site performance across studies of varied design and complexity.

**Results:**

Leveraging Covance's market leadership in central laboratory services, we have assembled a database of operational metrics that spans more than 14,000 protocols, 1400 indications, 230,000 unique investigators, and 23 million patient visits and represents a significant fraction of all clinical trials run globally in the last few years. By analyzing this historical data, we are able to assess and compare the performance of clinical investigators across a wide range of therapeutic areas and study designs. This information can be aggregated across trials and geographies to gain further insights into country and regional trends, sometimes with surprising results.

**Conclusions:**

The use of operational data from Covance Central Laboratories provides a unique perspective into the performance of clinical sites with respect to many important metrics such as patient enrollment and retention. These metrics can, in turn, be used to guide operational planning and site selection for new clinical trials, thereby accelerating recruitment, improving quality, and reducing cost.

## 1 Introduction

The soaring costs and declining productivity of drug development has intensified interest in tools and technologies that can improve the efficiency of clinical trials. From an operational standpoint, the goal is to complete the study as quickly as possible with as few sites as possible. The number of quality of the sites are important determinants of trial success. Fewer sites reduce logistical complexity and higher-quality sites minimize unnecessary delays in recruiting patients and successfully completing the protocol. Both are important value drivers, as they impact cost-per-patient and time-to-market, thus extending the drug's patent lifespan, the sponsor's return on investment, and the societal benefit of bringing important new therapies to patients in need.

The patient interfacing part of a clinical trial is conducted at independent medical institutions, such as university research centers, hospitals and doctors' offices. Because these independent sites are responsible for patient recruitment and engagement, they have a profound effect upon the number and rate at which patients are screened, enrolled and retained in a clinical trial, and ultimately upon the timeline for completing the study [1]. In addition to keeping the patients engaged and preventing patient drop-out, high performing sites can also increase the availability of data and the probability that a statistically significant therapeutic effect can be demonstrated at the end of the trial [1,2].^1^

Thus, the selection of high quality clinical sites during the planning phase of a trial is critical to its success, and improved methodologies to enable this process are of great interest to pharmaceutical companies and clinical contract research organizations (CROs). As a market leader in central laboratory testing and clinical trial management services, it has been our experience that the strongest predictor of a site's future performance is its historical record. Sites that have performed well in the past also tend to do well in the future. While this insight seems obvious, acting upon it is operationally difficult for two reasons. The first is the lack of data. Normally a pharmaceutical company or CRO will only have visibility into their own trials and not those sponsored by other companies and institutions, thus limiting their ability to obtain a sufficient volume of historical information to make robust assessments. The second challenge stems from the fact that the complexity of the clinical protocol itself can have significant impact upon patient recruitment and retention, making it difficult to compare investigators and sites that have not worked on the same trial. Like most “big data” analyses, the challenge is not so much the collection and aggregation of the data, but finding ways to analyze data that have been collected under significantly different assumptions and conditions; clinical trial data falls squarely into that category.

As a company with a market leading laboratory division that conducts clinical laboratory testing for more than 40% of the outsourced clinical trials in the world, Covance has assembled the most comprehensive database in the pharmaceutical industry, spanning more than 13,000 protocols, 1,400 clinical indications, 230,000 investigators, and 23 million patient visits. To enable communication with our clients and clinical sites and to ensure that the laboratory results can be effectively integrated with other clinical trial data, the laboratory samples are labeled with metadata such as anonymized patient identifiers, investigator names and addresses, sample collection and shipment dates, etc. While this information is captured primarily for operational purposes, we hypothesized that it could also be repurposed to compute site performance metrics such as patient enrollment, screen failure rates, drop-out rates, and other site quality characteristics. More importantly, since the central laboratory service is rarely changed during the course of the trial in order to minimize variability, the data that we collect is complete and consistent: if the laboratory part of a trial is conducted by Covance, all clinical test results for all patient visits across the world for that trial are captured and recorded through our systems. This allows us to develop reliable performance metrics and insights at the individual site and protocol levels, which in turn enable us to intelligently identify and prioritize high performing sites when planning a new trial.

From a design perspective, the inherent variability in trial complexity can be addressed in two complementary ways: a) by providing the user dynamic interfaces to explore the underlying data at any level of detail, and b) by normalizing and aggregating site performance in a way that minimizes these inherent differences and allows direct comparison of protocols of widely different designs and complexity. Here, we describe two different visualization approaches designed to address these needs. The first includes an interactive dashboard that allows project managers to drill down to individual sites' historical data relating to site performance and confirm or challenge their intuitions about each site's likely future performance. The second is a way of compressing this information into a single plot that offers unique insights into relative performance and aggregate trends. These two types of visualizations are highly complementary in that they render the information at different levels of granularity, and both have proven their utility in our clinical trial planning efforts.

While it may be tempting to eschew the use of interactive visualization in lieu of optimization algorithms that pick the “best” sites [3], it has been our experience that a great number of additional factors that cannot be easily quantified also play a role in determining whether a site is ultimately selected. These factors are often based on the individual study managers' intuition and prior experience working with the sites. It is generally accepted that a tool that allows an astute user to interact with the data yields better overall outcomes when the data supporting the selection decision is incomplete or qualitative. Furthermore, it has been shown in numerous cases that human intuition married to meaningful visualizations can lead to more optimal outcomes than a purely computational solution [4]. The visualizations described in this work are accordingly tailored to their target audience.

## 2 Methods

The fundamental hypothesis underlying these visualizations is that the metadata associated with the laboratory kits that we receive from the different clinical sites allow us to reconstruct the complete schedule of patient visits on an anonymized basis, and that these patient visits offer insights into the operational performance of their respective sites. Currently, each kit contains an anonymized patient identifier, a trial identifier, the date in which the sample was collected, and the investigator's name and address, which are important for shipping purposes. This allows us to reconstruct the visit schedule of each patient and associate him/her with a particular investigator and trial. Furthermore, regulatory requirements stipulating that laboratory tests associated with patient safety must be processed within 48 h from sample collection allow us to associate each laboratory kit to a specific patient visit with a high degree of precision. Because safety testing is an integral part of every clinical trial, this assumption is generalizable across the all phases, therapeutic areas and clinical indications.

More specifically, the first kit registered for a given patient in a given trial at a given site marks the time that this patient was first screened for that trial. When the second kit arrives for that same patient, same site and same trial, we can assume that this patient was enrolled in the trial (exceptions such as duplicate screening do exist, but are generally rare). Subsequent kit shipments trace the remaining patient visits in a similar fashion. Further, if a patient has fewer kits than expected, we can safely conclude that the patient has been terminated early. Therefore, by counting the number of different patient identifiers associated with a site, we can determine the number of patients screened. By counting the number of different patient identifiers with two or more kits, we can determine the number of patients enrolled. And by counting the number of kits per patient, we can determine which patients followed the visit schedule and which did not. Finally, by computing the time difference between the first kit of the first patient and the last kit of the last patient, we can estimate the length of time that the site was open. This provides us with robust metrics of clinical site performance, which can be further annotated by additional attributes, such as therapeutic area, clinical indication, geography, etc.

**Fig. 1:**
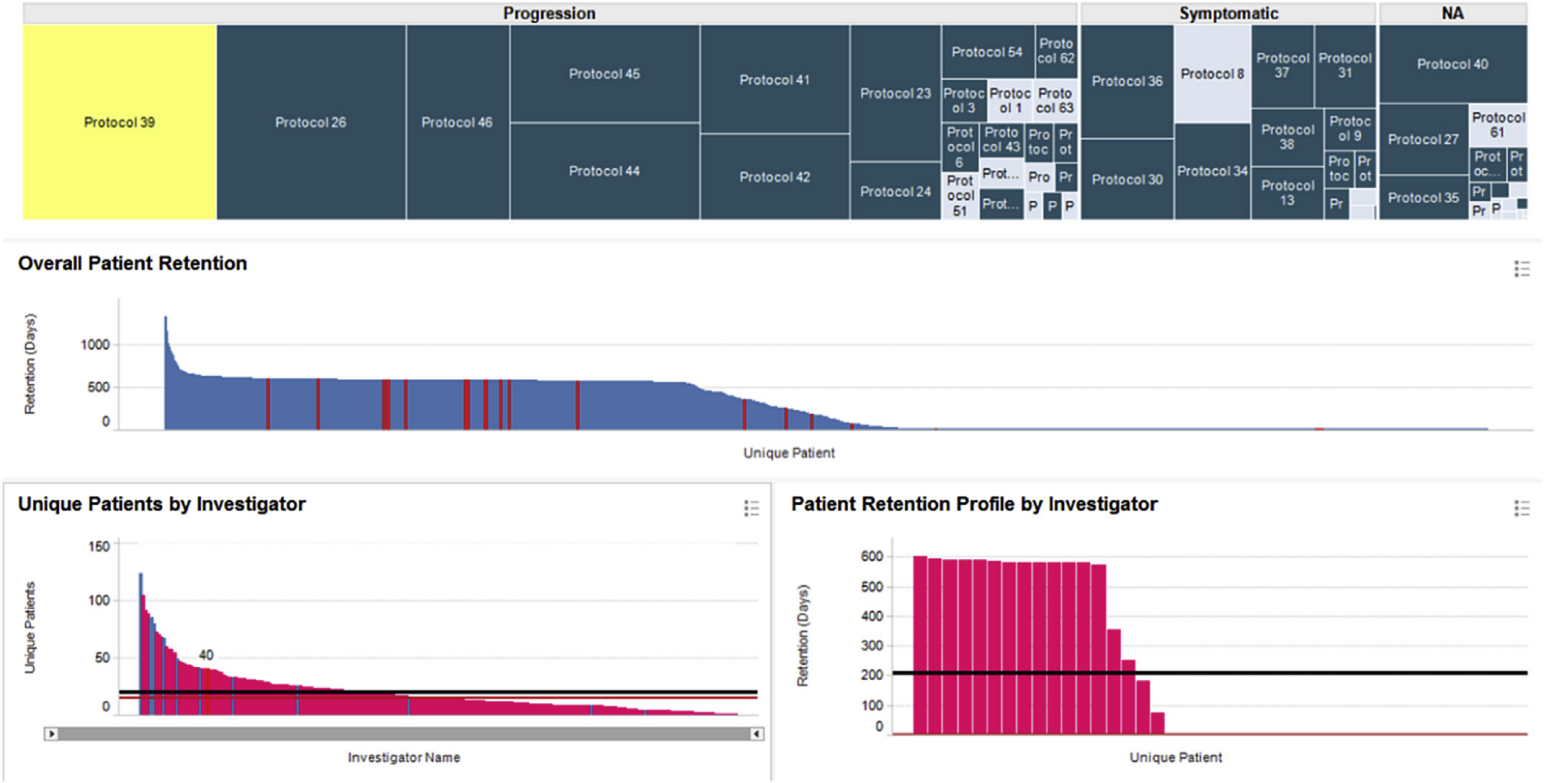
Protocol-centric view of investigator performance for all Alzheimer's trials conducted through our central labs. Protocols in the treemap are grouped based on whether the therapy is disease modifying, symptomatic, or unspecified.

**Fig. 2:**
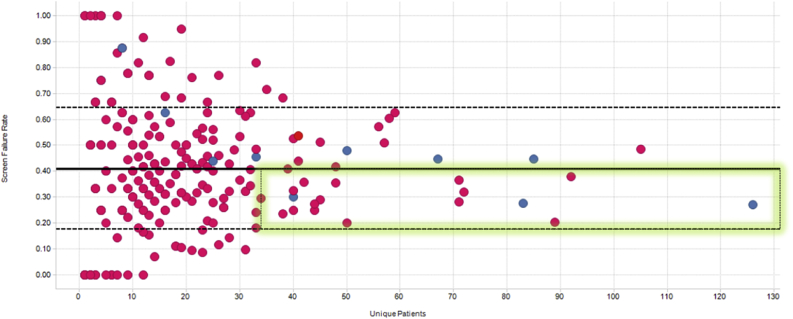
Screen failure rates vs number of patients recruited. Covance in-network and out-of-network investigators are shown in blue and pink circles, respectively. The mean and inter-quartile ranges are indicated by the solid and dashed lines. The best performing investigators who show the largest enrollment counts and lowest screen failure rates are highlighted by the green rectangle.

**Fig. 3:**
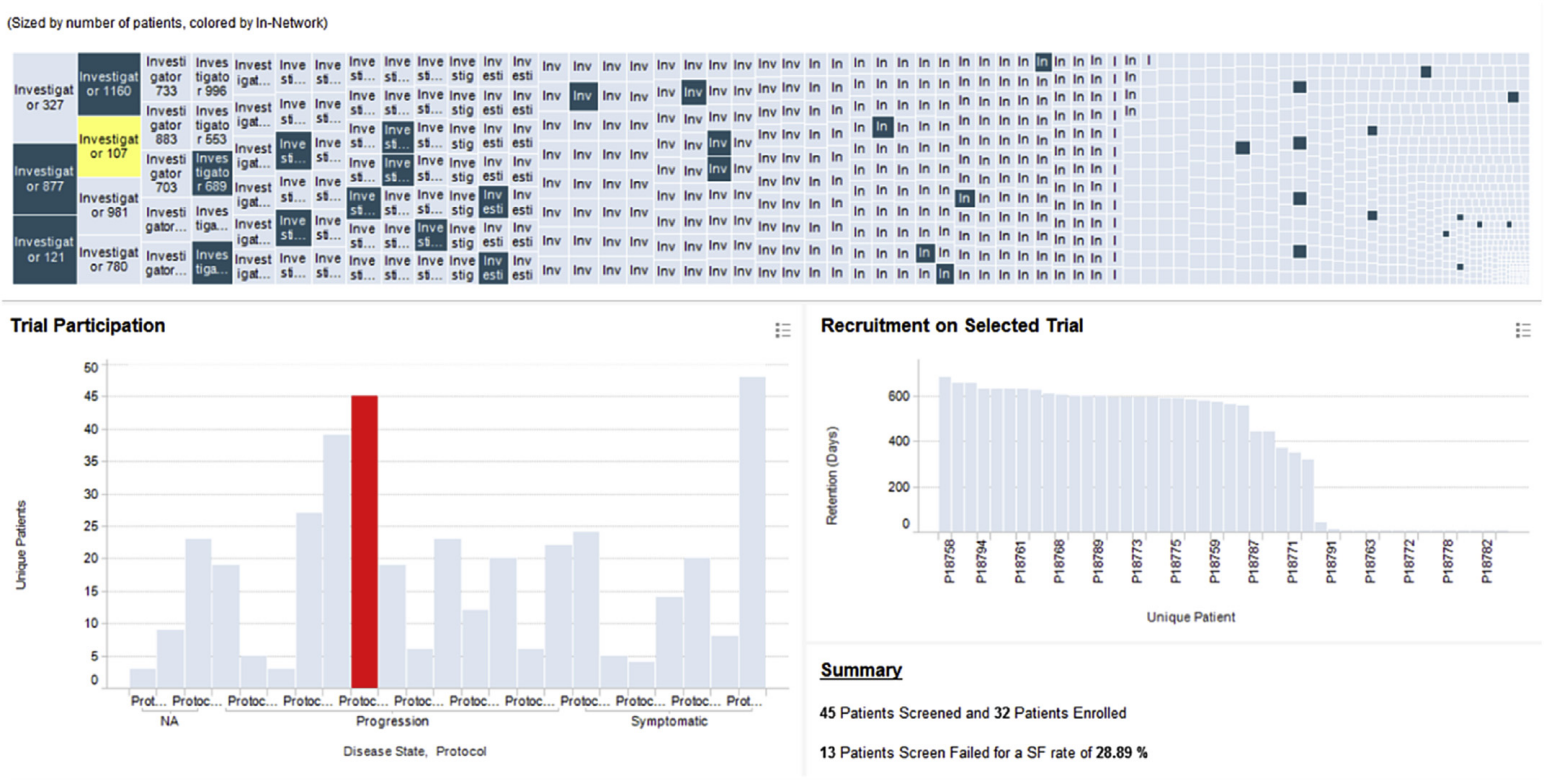
Investigator-centric view of Alzheimer's enrollment data. Dark and light blue rectangles in the treemap represent in-network and out-of-network investigators, respectively.

**Fig. 4:**
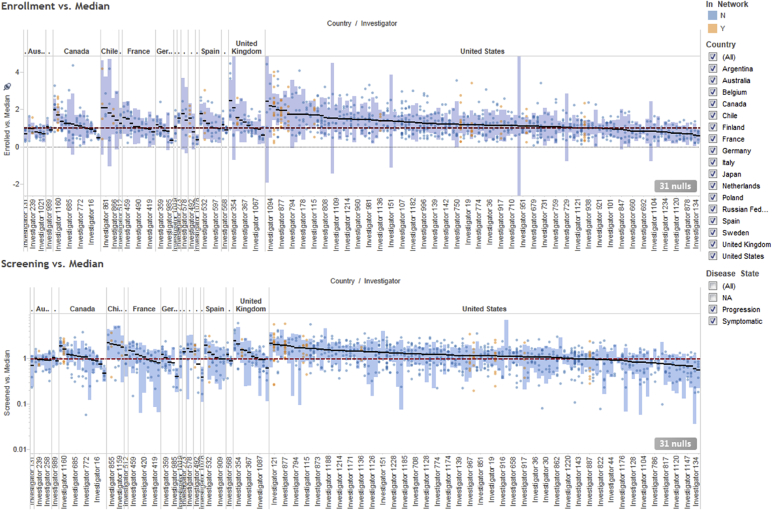
Normalized screening (bottom) and enrollment (top) investigator performance, organized by country.

**Fig. 5:**
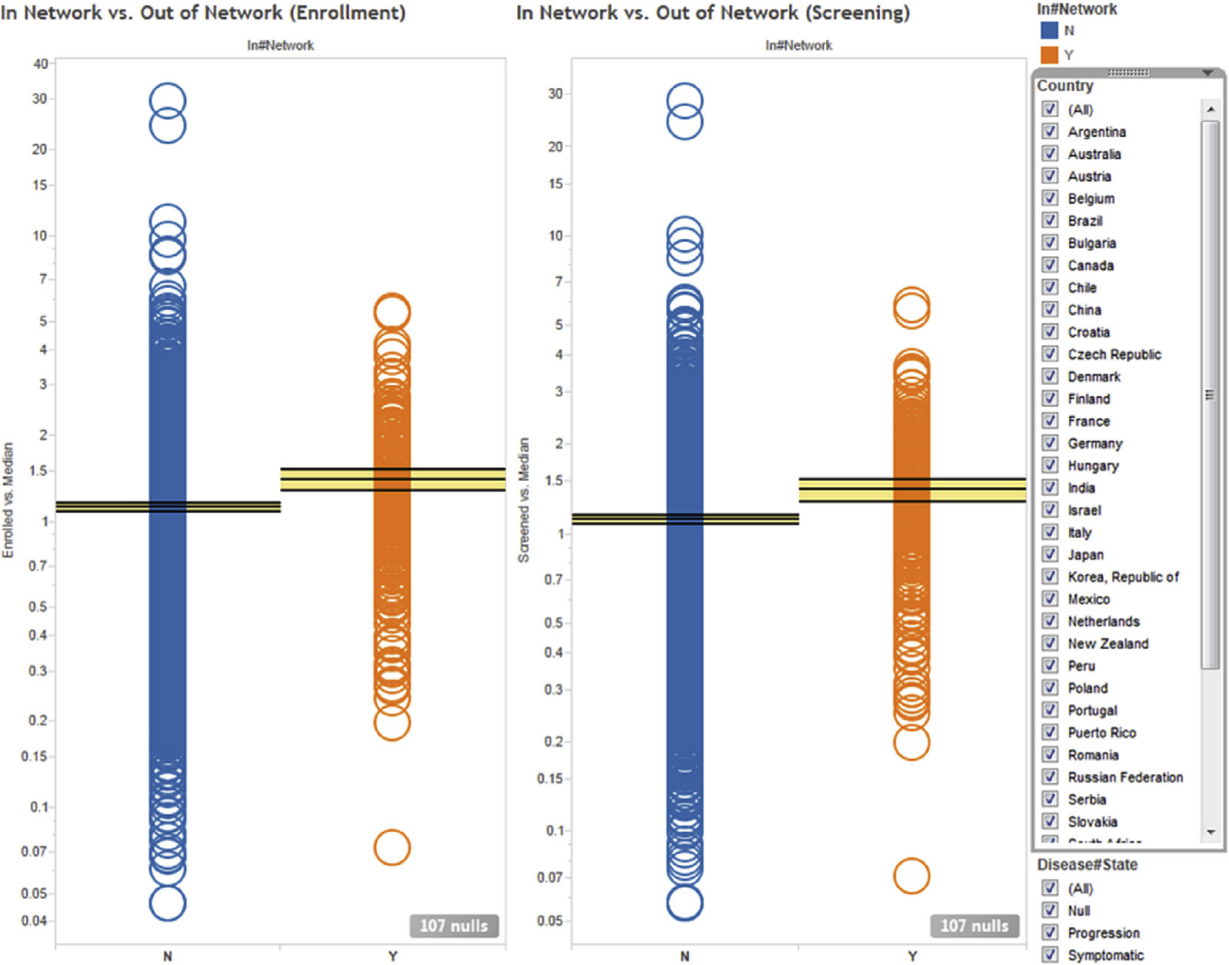
Normalized screening (right) and enrollment (left) investigator performance averaged over all clinical protocols they participated in. Each circle represents a unique investigator. Orange and blue circles indicate in-network and out-of-network investigators, respectively. The horizontal lines and yellow bands indicate the mean and 95% confidence intervals, respectively.

Our initial efforts at exploring this data relied upon the interactive visualization capabilities of Spotfire [5] and Tableau [6]. Fig. 1, Fig. 2, Fig. 3, Fig. 4, Fig. 5 illustrate representative displays exploring various aspects of patient screening, enrollment and retention. These plots are general in nature and can be generated with many different data visualization tools. The novelty of our approach lies not in the specific graphing components, but in the utilization of a novel dataset to glean insights into the operational performance of clinical sites. We believe that this dataset is unique in the industry and dwarfs many other sources of data, both commercial and public, in depth and breadth.

Raw performance metrics such as enrollment and drop-out rates vary widely among trials, even for a single site. They are affected as much by the site's underlying operational quality as by the design of the protocol itself. A high performing site is typically defined as one that enrolls a lot of patients and retains the majority of them through the completion of the study. However, this definition falls short when comparing sites that have participated in two different trials because the stringency of the inclusion/exclusion criteria could be markedly different. For instance, a site could have a low recruitment rate either because it is operationally mediocre or because the trials that it participated in were inherently harder to recruit for (e.g., the available patient populations were intrinsically smaller). One way to minimize the variance due to protocol differences is to only compare a site to its peers within the same trial. We devised a normalization strategy where each site's performance in a given trial is compared to the median performance of that trial via a simple ratio. If a site enrolls twice the median number of patients, their normalized performance is 2; if it enrolls half the median number of patients, their normalized performance is 0.5. We chose the median rather than the mean to account for the fact that there is a large fraction of sites that do not enroll any patients and there are also professional sites that aggregate the results of multiple smaller sites leading to inflated patient counts.

For the examples illustrated below, we used the data for all phase 2–3 Alzheimer's Disease trials that utilized our central laboratory services in the last 10 years. This consisted of 58 trials encompassing nearly 1,200 clinical sites, far more than any individual CRO or pharmaceutical company would have access to.

## 3 Results

The treemap [7] shown in the top panel in Fig. 1 organizes the data by protocol and contains a rectangle for each of the 58 Alzheimer's trials which shipped samples to our central laboratories. The protocols are divided into three main branches depending on whether the therapy was disease modifying, symptomatic or unspecified. The area of each rectangle is proportional to the number of patients recruited in that trial.

When the user selects a protocol (or multiple protocols) in the treemap, the bar charts in the middle and lower left panes are updated to respectively display the patients and investigators who participated in it. The middle pane shows the retention of each patient in the trial, defined as the number of days between the first and the last kit registered for that patient. Each patient is represented by a distinct bar whose height is proportional to the number of days s/he remained on the trial. The bars are sorted in descending order of retention to enable quick visual assessment of the overall screening and drop-out rates. For the specific protocol highlighted in yellow, we can see that ∼45% of the patients were screen failures (they were retained for only one day), ∼15% dropped out for various reasons, ∼40% followed the complete visit schedule, and ∼2% required additional unscheduled visits.

The bottom left pane displays the number of patients screened by each investigator participating in the selected trial, again sorted in descending order, with the median and average shown by the black and red horizontal lines, respectively. When selecting an individual investigator, the user can see their overall patient retention profile on the bottom right panel. In this specific case, we see that the selected investigator has recruited 17 patients, four of whom dropped out and none required an unscheduled visit. More importantly, we see an unusually high screen failure rate (SFR) of >50%. Both very low and very high SFRs are worrisome: no or few screen failures may suggest a lack of oversight by the site, whereas high SFRs impose an additional cost burden on the program. The ideal investigators are those who recruit a lot of patients and have SFRs at or just below the study's mean SFR, as highlighted by the green rectangle in Fig. 2. This view is important because of the financial incentives associated with clinical trials where study sites are paid by the number of patients enrolled and not by how well those patients meet the protocol's eligibility criteria. Sites with historically abnormal SFRs can either by excluded from participating in new studies or be subject to additional scrutiny once they begin enrolling patients.

An alternative investigator-centric view of the same information is illustrated in Fig. 3. Here, the treemap at the top contains a rectangle for each of the ∼1,200 investigators who participated in the 58 Alzheimer's trials. The size of each rectangle is proportional to the number of patients screened by the corresponding investigator across all Alzheimer's protocols. When the user clicks on a specific investigator, the plot at the bottom left shows the protocols that the selected investigator participated in and the number of patients s/he screened in each protocol. When the user clicks on a particular protocol, the plot on the right shows the corresponding patient retention profile.

The treemap in Fig. 3 is color coded to indicate whether an investigator is in Covance's preferred network (dark blue) or not (light blue). In-network investigators are experts with extensive experience in the therapeutic area of interest, an established relationship with Covance, and have been identified by study managers as having above-average performance in prior engagements. As seen from Fig. 3 (and further articulated below), while our in-network investigators are generally good recruiters, they represent a small fraction of all possible investigators whose performance we can infer through the metadata from our central laboratory operations. These dynamic, linked visualizations with drill-down and detail-on-demand capabilities allow project planners to rapidly explore the data and select strong investigator candidates when planning a new clinical trial.

Another insight that one can draw from Fig. 3 is that the performance of each investigator can vary greatly from protocol to protocol. Therefore, rather than calculating simple aggregations such as the mean or median of a site's performance over multiple trials, we have elected to first normalize site performance as discussed above. This normalization strategy allows us to represent site performance independent of protocol difficulty and enables meaningful and information-rich visualizations, such as the one illustrated in Fig. 4.

The two panels in Fig. 4 represent the normalized screening (bottom) and enrollment (top) performance of a given site in a given trial. Each small filled circle represents a distinct trial/site combination (to ensure statistical validity, only sites that participated in 3 or more Alzheimer's trials are included). Orange and blue circles denote in-network and out-of-network investigators, respectively. The horizontal red dotted line crossing the y-axis at 1 represents the median screening/enrollment performance. Values above that line indicate better-than-median performance, whereas values below that line indicate worse-than-median performance. The small black horizontal bars represent the overall normalized performance of each site averaged over all the trials that they participated in, and the long blue vertical bars represent the corresponding 95% confidence intervals. Grouping the investigators by various attributes such as country of origin allows visual assessment of the impact of these attributes upon performance.

In Fig. 4, the sites are split by country, allowing the user to assess performance at both the individual site and country levels. For example, the majority of the US sites are better than average compared to their peers in other countries, which is unsurprising given the fact that the majority of Alzheimer's trials have a significant US component that builds up the sites' experience and leads to sponsors using the best of them in more and more studies. However, counterintuitively, we find that all the sites in Australia are below average. For a country with an advanced western-style medical system, one would assume that Australia would be on par with the United States and other European countries, which have a mix of high and low performing sites. This is clearly not the case and, while surprising, it does confirm the intuition of individuals who have done extensive work on Alzheimer's disease in Australia.^2^ Conversely, the majority of the sites in the UK performed significantly better than average. Additionally, certain South American countries such as Chile show consistently high performance, comparable to Western countries. Thus, through these visualizations we are able to assess not only which individual sites perform consistently better than their peers, but also which countries as a whole provide fertile grounds for Alzheimer's clinical research.

As previously stated, the data can be aggregated and analyzed across any desirable dimension. Fig. 5 illustrates an alternative view where the sites are split by whether they were part of Covance's network of preferred clinical sites (in-network vs. out-of-network, as previously described). This plot shows that Covance's preferred investigators screen and recruit on average ∼25% more patients than sites outside of Covance's network. While this is comforting, we also see evidence that some of the sites that had initially been selected perform poorly, and thus are candidates for either replacement or remediation.

## 4 Conclusions

By utilizing the data derived from Covance's central laboratory operations, we are able to provide in-depth visibility into the historical operational performance of investigational sites from a large number of clinical trials. Furthermore, with basic normalization, we can derive robust metrics that account for differences in study protocols and enable direct comparison of site performance across trials of widely varying designs. Coupled with interactive data visualization techniques, these metrics can help study teams improve country allocation, optimize site networks, and set sensible enrollment targets. One of the strengths of interactive data visualization is that it allows users who are not formally trained in data mining, informatics or other related quantitative disciplines to apply and test their intuitions while still maintaining a data-driven approach to decision making. In this work, we presented several visualizations that allow clinical trial planners to mine large volumes of historical enrollment data to identify and selectively target the highest performing investigators when designing a new trial. Country and site selection is one of the most critical operational decisions that can be made in the planning phases of a new trial, and has the greatest potential for improving the quality and reducing the time and cost of drug development. While this specific analysis focused on patient enrollment and Alzheimer's Disease, the approach is generalizable and can be applied to many other quality metrics and clinical indications.

## Appendix A Supplementary data

mmc1 mmc1

## Footnotes

1 It is important to note that we define “good” sites based solely on their operational performance and not on any clinical outcome measures. Given that the majority of experimental drugs fail in clinical trials, selecting sites that report treatment effects in their patient cohorts when there is no treatment effect in the wider population would be highly questionable. Of course, patient enrollment and retention are not the only relevant indicators of site quality. Other factors such as improper subject eligibility or discontinuation, underreporting of adverse events, excessive lab test cancellations and repeats, etc. are equally important. Some of these metrics can be derived from laboratory data, but others require access to a broader range of systems used for clinical trial management and oversight (CTMS, EDC, IRT, etc.). Given the insular nature of the pharmaceutical industry and the fragmentation of the clinical technology and services market, this information is not easily accessible at the scale and coverage described in the present work, and is typically confined within the walls of individual pharmaceutical companies and/or software and CRO vendors. Some cross-company initiatives, such as those sponsored by the TransCelerate consortium, attempt to break down these silos, but they are focused on basic operational capabilities, such as maintaining a shared repository of business contact information and a common portal for portal for engaging with investigative sites.

2 This work was originally carried out as part of a bid defense for a large, phase 3 Alzheimer's study with a large pharmaceutical sponsor with extensive experience in this therapeutic area. This result was notable to the sponsor team because, while many individuals had a “hunch” that Australia would not be able to meet their recruitment targets, they were unable to substantiate their intuition with hard data and convince their operational teams responsible for country and site selection to change the enrollment targets for that country.
